# Activity of cefiderocol against *Pseudomonas aeruginosa* from the USA and Europe (2020–2023) with difficult-to-treat resistance phenotype, including those nonsusceptible to recently developed β-lactam/β-lactamase inhibitor combinations: results from the SENTRY antimicrobial surveillance program

**DOI:** 10.1128/spectrum.02079-25

**Published:** 2025-10-13

**Authors:** John H. Kimbrough, Maura H. Karr, Sean T. Nguyen, Boudewijn L. M. DeJonge, Chris Longshaw, Miki Takemura, Yoshinori Yamano, Mariana Castanheira, Rodrigo E. Mendes

**Affiliations:** 1 Element Iowa City (JMI Laboratories) 138461, North Liberty, Iowa, USA; 2 Shionogi Inc. 71777 , Florham Park, New Jersey, USA; 3 Shionogi B.V., London, United Kingdom; 4 Shionogi & Co., Ltd., Osaka, Japan; Johns Hopkins University, Baltimore, Maryland, USA

**Keywords:** MBL, carbapenem resistance, DTR, IDSA, guidelines

## Abstract

Cefiderocol and the β-lactam/β-lactamase inhibitor (BL-BLI) combinations ceftazidime-avibactam (CAZ-AVI), imipenem-relebactam (IMI-REL), and ceftolozane-tazobactam (TOL-TAZ) are recommended for the treatment of difficult-to-treat resistance (DTR) *Pseudomonas aeruginosa* by IDSA guidelines. The activity of cefiderocol and the three BL-BLI combinations described above was examined against DTR *P. aeruginosa* and molecularly characterized subsets collected from the USA and Europe (2020–2023). Approximately 4% of isolates showed a DTR phenotype, and cefiderocol remained active against 98.1% (CLSI breakpoint) of these isolates, whereas the three BL-BLI combinations had susceptibilities of 52.3%–56.0%. A total of 63% of DTR isolates were nonsusceptible to ≥1 of these BL-BLI combinations, and cefiderocol was active against ≥95.5% (CLSI) of these isolates, regardless of the nonsusceptible phenotype(s) to these combinations. In contrast, susceptibilities of the BL-BLI combinations varied from 0.0% to 30.3%. Almost 20% of DTR isolates carried carbapenemases, mostly metallo-β-lactamases, represented by 14 different STs from 15 countries. Cefiderocol had MIC_50/90_ of 0.25/2 mg/L against these carbapenemase-carrying DTR isolates and 97.3% (CLSI) of the isolates were susceptible to cefiderocol, whereas <6% of these isolates were susceptible to any of the BL-BLI combinations. MIC_50/90_ of cefiderocol against the non-carbapenemase DTR subset was 0.12/1 mg/L, and 98.3% (CLSI) of the isolates were susceptible to cefiderocol, vs 63.7%–69.6% for the BL-BLI combinations. Cefiderocol demonstrated high activity against a large and contemporary collection of DTR *P. aeruginosa*, regardless of carbapenemase status. The three currently recommended BL-BLI combinations for treating certain *P. aeruginosa* infections demonstrated limited activity and high degree of cross-resistance.

**IMPORTANCE:**

Cefiderocol and the β-lactam/β-lactamase inhibitor (BL-BLI) combinations ceftazidime-avibactam (CAZ-AVI), imipenem-relebactam (IMI-REL), and ceftolozane-tazobactam (TOL-TAZ) are recommended for the treatment of difficult-to-treat resistance (DTR) *Pseudomonas aeruginosa* by IDSA guidelines. However, this study shows that cefiderocol demonstrated high activity against a large collection of *P. aeruginosa*, including DTR isolates. In contrast, the three currently recommended BL-BLI combinations had limited activity, especially against isolates carrying carbapenemase genes, and high degree of cross-resistance. Cefiderocol also sustained activity against a diverse array of isolates with various BL-BLI nonsusceptible phenotypes, and regardless of carbapenemase status. These findings support the use of cefiderocol as a first treatment option for infections caused by DTR *P. aeruginosa*.

## INTRODUCTION

*Pseudomonas aeruginosa* is an opportunistic pathogen with broad metabolic capabilities, a diverse array of virulence factors, and propensity to develop and/or acquire resistance to many currently available antibiotics. Of particular concern is the emergence of *P. aeruginosa* isolates resistant to third- and fourth-generation cephalosporins, carbapenems, and β-lactam/β-lactamase inhibitor (BL-BLI) combinations. Acquired mobile genetic elements encoding extended-spectrum β-lactamase (ESBL) and carbapenemase genes are important resistance mechanisms to broad-spectrum β-lactam agents in *P. aeruginosa*; however, resistance typically results from mutational events affecting intrinsic mechanisms of cellular homeostasis (1). These mutations frequently coalesce around three main mechanisms promoting resistance: mutation and/or derepression of the chromosomal *ampC* (*Pseudomonas*-derived cephalosporinase, PDC) (2–5), increased drug efflux through overexpression of drug-specific efflux pumps (6, 7), and limitation of membrane permeability (8–10).

Many of the intrinsic resistance mechanisms in clinical *P. aeruginosa* are present in globally distributed multidrug-resistant (MDR) clones, including those from multilocus sequence type (MLST) 111 (11, 12), ST235 (11, 13, 14), and ST175 (12, 15), among others. These resistance mechanisms may play a role in those infections caused by *P. aeruginosa* with “difficult-to-treat resistance (DTR)” phenotypes, which are nonsusceptible to all frontline treatment options (16). Moreover, acquired ESBL and carbapenemase-encoding genes are increasingly identified in clinical *P. aeruginosa* isolates (12, 17, 18), underscoring the need for continued surveillance and development of new treatment options.

In general, two main strategies have been employed thus far to combat the ongoing obsolescence of the current options against infections caused by *P. aeruginosa*: developing novel therapeutic agents and rescuing traditional β-lactam or carbapenem agents by combining them with new BLI agents. Cefiderocol, a catechol-cephalosporin conjugate, uses intrinsic iron-transport systems to gain entry into the periplasm space where it binds and inhibits penicillin-binding protein 3 (PBP3), thereby disrupting peptidoglycan biosynthesis (19, 20). Surveillance and mechanistic studies have demonstrated cefiderocol’s broad activity against phenotypically resistant (e.g., MDR, DTR) (21–23) and genotypically diverse *P. aeruginosa* strains, including those harboring ESBL, serine carbapenemase (21), metallo-β-lactamase (MBL) genes (21), and alterations affecting the activities and/or expression of PDC (24), OprD, and efflux pumps (19, 23). Moreover, effective use of cefiderocol therapy has been reported in treating infections caused by *P. aeruginosa* exhibiting various resistance profiles (25–27).

The primary BL-BLI combinations currently used in the treatment of *P. aeruginosa* infections include piperacillin-tazobactam, ceftolozane-tazobactam (TOL-TAZ), ceftazidime-avibactam (CAZ-AVI), and imipenem-relebactam (IMI-REL). Despite their clinical utility against numerous phenotypic classifications of *P. aeruginosa* (28, 29), these combinations are limited in their activity against those strains possessing the common mutational resistance mechanisms described above, including efflux increase (30–32), OprD loss (31, 32), and derepressed expression of the PDC with mutations affecting substrate profile and/or inhibitor efficacy (2, 33, 34). Most importantly, none of these combinations are effective against isolates harboring class B MBL genes (11, 29).

In 2024, the Infectious Disease Society of America issued updated guidance for treating DTR *P. aeruginosa* in cases of uncomplicated cystitis, pyelonephritis, and complicated urinary tract infections when antimicrobial susceptibility testing results are available (16). For these infection types, TOL-TAZ, CAZ-AVI, IMI-REL (described herein as the three IDSA-recommended BL-BLI combinations), and cefiderocol are the preferred options for treatment owing to the large amount of clinical trial, surveillance, and observational studies. For infections outside the urinary tract, IDSA recommends these three BL-BLIs as primary treatment options with cefiderocol as an alternative option when these BL-BLI are ineffective.

In this study, the activity of cefiderocol and IDSA-recommended BL-BLI combinations was assessed against *P. aeruginosa* isolates and a subset showing a DTR phenotype collected as part of the SENTRY Antimicrobial Surveillance Program during 2020–2023. We further examined the frequency of isolates nonsusceptible to these BL-BLI agents and molecularly characterized these isolates to define potential β-lactamases underlying the presentation and distribution of these phenotypes among currently circulating *P. aeruginosa* clinical isolates in hospitals across the USA, Europe (EUR), Turkey, and Israel.

## MATERIALS AND METHODS

### Isolate collection

A total of 9,572 *P*. *aeruginosa* clinical isolates recovered from various infections during 2020–2023 across 19 countries from North America (USA; 36 hospitals; *n* = 4,400) and Europe and adjacent regions (43 hospitals; *n =* 5,172) were included (Table S1). Isolates were derived from bloodstream infections (14.6%), intra-abdominal infections and other sites (4.3%), pneumonia in hospitalized patients (52.9%), skin and soft tissue infections (19.1%), and urinary tract infections (9.1%) (Table S1). Bacterial species were identified by the submitting laboratory and confirmed by Element Iowa City (JMI Laboratories) using standard microbiology methods and matrix-assisted laser desorption-time of flight mass spectrometry (Bruker Daltonics, Bremen, Germany).

### Susceptibility testing

Broth microdilution method testing was performed according to established CLSI protocols (35). Cation-adjusted Mueller-Hinton broth (CAMHB) was used for generating MIC results for comparator agents, whereas iron-depleted CAMHB was used for cefiderocol (35). Fixed concentrations of 4 mg/L were used for avibactam, tazobactam, and relebactam in combination with their respective β-lactam partners (35). MIC results for cefiderocol were interpreted following the CLSI, EUCAST, and FDA criteria (36, 37, 38); all other comparator agents were interpreted according to CLSI. A DTR phenotype was considered when isolates exhibited nonsusceptible (CLSI breakpoints) MIC results to piperacillin-tazobactam, ceftazidime, cefepime, aztreonam, meropenem, imipenem, ciprofloxacin, and levofloxacin (16).

### Screening of resistance mechanisms

DTR isolates were sequenced using Illumina short-read sequencing and *in silico* screened for β-lactamase genes. Total genomic DNA was prepared using the KingFisher Cell and Tissue DNA kit (ThermoFisher Scientific, Waltham, MA, USA) or the MagMax DNA Multi-Sample Ultra 2.0 extraction kit (ThermoFisher) on a KingFisher Flex Magnetic Particle Processor (ThermoFisher). DNA libraries were constructed using either the Nextera XT library construction protocol and index kit or the Illumina DNA prep (Illumina, San Diego, CA, USA) with sequencing performed on either a MiSeq Sequencer with a MiSeq Reagent Kit v3 (600 cycles) or a NextSeq 1000 Sequencer using NextSeq1000/2000 P2 Reagents (300 cycles).

FASTQ format files for each sample set were assembled independently using the *de novo* assembler SPAdes, using the as-then current version, with K-values of 21, 33, 55, 77, and 99 plus “careful mode” on to reduce the number of mismatches (39). This process produced a FASTA format file of contiguous sequences with the best N50 value. An in-house proprietary bioinformatics pipeline and a curated resistance gene database based on the National Center for Biotechnology Information Bacterial Antimicrobial Resistance Reference Gene Database (https://www.ncbi.nlm.nih.gov/bioproject/313047) were used for the *in silico* screening of β-lactamase genes. All genes were used as queries to align against the target assembled sequences. MLST was determined by extracting the relevant alleles from the sequencing data for each isolate and querying against the database available at pubmlst.org. When no type was available (novel-types, single or multilocus variants), “-like” was appended to the nearest available ST.

The phylogeny of 70 DTR isolates nonsusceptible to all three IDSA recommended BL-BLI combinations and carrying carbapenemases was constructed by creating a core genome MSLT (cgMLST; 3,867 loci) for each isolate using chewBBACA v3.2.0 (40) based on the scheme for *P. aeruginosa* available at cgmlst.org (41). A multiple genome alignment was performed using MUSCLE v3.8.11 (42), and an unrooted maximum likelihood tree was created with FastTree v2.2.11 using default settings (43). The maximum likelihood tree and associated metadata was curated with iTol v6.8.2 (44).

## RESULTS

Overall, cefiderocol (MIC_50/90_, 0.12/0.25 mg/L) was active against the entire *P. aeruginosa* collection, and 98.6%–99.8% of isolates were susceptible under the current CLSI, EUCAST, or FDA interpretive criteria (Table 1), regardless of geographic region (Tables S2 and S3. CAZ-AVI (96.1% susceptible), IMI-REL (96.5%), and TOL-TAZ (95.9%) were also active against the entire collection (Table 1). Susceptibilities obtained for these three IDSA- recommended BL-BLI agents (CAZ-AVI, IMI-REL, and TOL-TAZ) were slightly lower in EUR (95.6%, 95.5%, and 94.7%, respectively) compared to those in the USA (96.7%, 97.6%, and 97.4%, respectively) (Tables S2 and S3).

**TABLE 1:** Activity of cefiderocol and comparator agents tested against DTR and non-DTR *P. aeruginosa* collected from US and European medical centers (2020–2023)

		% susceptible^*b*^					
		FDC^*a*^			CAZ-AVI^*a*^	IMI-REL^*a*^	TOL-TAZ^*a*^
Susceptibility profile^*a*^ (*n*; %)	MIC_50/90_	CLSI	EUCAST	FDA		CLSI	
All isolates (9,572; 100)	0.12/0.25	99.8	99.5	98.6	96.1	96.5	95.9
DTR^*c*^ (377; 3.9)	0.12/2	98.1	96.8	88.9	52.3	56.0	54.4
BL-BLI-NS^*d*^ (238; 63.1)	0.25/2	97.5	95.8	85.7	24.4	30.3	27.7
CAZ-AVI-NS (180; 47.7)	0.25/2	97.2	95.0	83.9		29.4	25.0
IMI-REL-NS (166; 44.0)	0.25/2	97.0	95.8	86.7	23.5		22.9
TOL-TAZ-NS (172; 45.6)	0.25/2	96.5	94.8	82.0	21.5	25.6	
CAZ-AVI/IMI-REL-NS (127; 33.6)	0.25/2	96.1	94.5	84.3			13.4
CAZ-AVI/TOL-TAZ-NS (135; 35.8)	0.25/2	96.3	94.1	79.3		18.5	
IMI-REL/TOL-TAZ-NS (128; 34.0)	0.25/2	96.1	94.5	84.4	14.1		
CAZ-AVI/IMI-REL/TOL-TAZ-NS (110; 29.2)	0.25/2	95.5	93.6	81.8			

^*a*^ FDC, cefiderocol; CAZ-AVI, ceftazidime-avibactam; IMI-REL, imipenem-relebactam; TOL-TAZ, ceftolozane-tazobactam; DTR, difficult-to-treat, NS, nonsusceptible.

^*b*^ CLSI (2025), EUCAST (2025), and the FDA breakpoints for cefiderocol applied. CLSI (2025) breakpoints applied for comparator agents.

^*c*^ Isolates exhibiting nonsusceptible MIC results (CLSI) to piperacillin-tazobactam, ceftazidime, cefepime, aztreonam, meropenem, imipenem-cilastatin, ciprofloxacin, and levofloxacin.

^*d*^ Isolates that were nonsusceptible to ≥1 of the following BL-BLI combination agent: ceftazidime-avibactam, imipenem-relebactam, and/or ceftolozane-tazobactam.

A total of 3.9% (377/9,572) of isolates showed a DTR phenotype, with slightly higher proportions among EUR isolates (4.3%) compared to USA (3.5%). In addition, the proportion of DTR isolates in Eastern and Western EUR was 7.6% and 2.9%, respectively (Table S1). Against this group of DTR *P. aeruginosa*, 88.9%–98.1% of isolates were susceptible to cefiderocol (MIC_50/90_, 0.12/2 mg/L) (Table 1). Similar activities were observed for cefiderocol against DTR isolates from USA (90.3%–98.7% susceptible; MIC_50/90_, 0.12/1 mg/L) and EUR (87.9%–97.8% susceptible; MIC_50/90_, 0.12/2 mg/L) (Tables S2 and S3). In contrast, CAZ-AVI (MIC_50/90_, 8/>32 mg/L), IMI-REL (MIC_50/90_, 2/>8 mg/L), and TOL-TAZ (MIC_50/90_, 4/>16 mg/L) showed susceptibilities of 52.3%–56.0% against the DTR population (Tables 1 and 2). Other comparator agents, including the aztreonam-avibactam combination (MIC_50/90_, >16/>16 mg/L), were not active against DTR isolates, except for colistin (99.7% susceptible) (Table 2).

**TABLE 2:** Activity of cefiderocol and comparator BL-BLI combinations against DTR *P. aeruginosa* isolates collected in US and European medical centers (2020–2023)

Organism group Antimicrobial agent	MIC (mg/L)			CLSI^*a*^		EUCAST^*a*^		FDA^*a*^	
	50%/90%		Range	%S	%R	%S	%R	%S	%R
Non-DTR (9,195)									
Cefiderocol	0.12	0.25	≤0.004 to 32	99.8	0.1	99.6	0.4	99.0	0.4
Ceftazidime-avibactam	2	4	≤0.015 to >32	97.9	2.1	97.9	2.1	97.9^*b*^	2.1
Imipenem-relebactam	0.25	1	≤0.03 to >8	98.1	1.1	98.1	1.9	98.1^*b*^	1.1
Ceftolozane-tazobactam	0.5	2	≤0.12 to >16	97.6	1.5	97.6	2.4	97.6^*b*^	1.5
Aztreonam-avibactam	4	16	≤0.03 to >16						
Ceftazidime	2	16	0.06 to >32	86.4	9.9	^*c*^	13.6	86.4	13.6
Cefepime	2	16	0.06 to >32	89.4	3.2	^*c*^	10.6	89.4	10.6
Meropenem	0.5	8	≤0.015 to >32	83.6	10.8	83.6^*d*^	5.9	83.6^*b*^	10.8
Imipenem	1	8	≤0.12 to >8	81.6	14.4	^*c*^	14.4	81.6^*b*^	14.4
Aztreonam	8	>16	0.06 to >16	75.0	15.0	^*c*^	15.0	75.0^*b*^	15.0
Piperacillin-tazobactam	4	64	≤0.06 to >128	82.6	11.7	*^c^*	17.4	72.4	17.4
Ciprofloxacin	0.12	2	≤0.008 to >4	84.1	11.2	^*c*^	15.9	84.1^*b*^	11.2
Levofloxacin	0.5	4	≤0.015 to >32	75.5	14.7	^*c*^	14.7	75.5^*b*^	14.7
Colistin	0.5	1	≤0.06 to >8		0.4	99.8	0.2		
DTR^*e*^ (377)									
Cefiderocol	0.12	2	≤0.004 to >64	98.1	1.1	96.8	3.2	88.9	3.2
Ceftazidime-avibactam	8	>32	1 to >32	52.3	47.7	52.3	47.7	52.3^*b*^	47.7
Imipenem-relebactam	2	>8	0.25 to >8	56.0	29.4	56.0	44.0	56.0^*b*^	29.4
Ceftolozane-tazobactam	4	>16	1 to >16	54.4	34.7	54.4	45.6	54.4^*b*^	34.7
Aztreonam-avibactam	>16	>16	0.5 to >16						
Ceftazidime	>32	>32	16 to >32	0.0	86.7	^*c*^	100.0	0.0	100.0
Cefepime	32	>32	16 to >32	0.0	57.3	^*c*^	100.0	0.0	100.0
Meropenem	16	>32	4 to >32	0.0	94.2	0.0^*d*^	74.3	0.0^*b*^	94.2
Imipenem	>8	>8	4 to >8	0.0	93.1	^*c*^	93.1	0.0^*b*^	93.1
Aztreonam	>16	>16	16 to >16	0.0	81.4	^*c*^	81.4	0.0^*b*^ ^b^	81.4
Piperacillin-tazobactam	128	>128	32 to >128	0.0	88.9	^*c*^	100.0	0.0	100.0
Ciprofloxacin	>4	>4	1 to >4	0.0	85.1	*^c^*	100.0	0.0^*b*^	85.1
Levofloxacin	16	>32	2 to >32	0.0	95.8	*^c^*	95.8	0.0^*b*^	95.8
Colistin	0.5	1	≤0.06 to >8		0.3	99.7	0.3		

^*a*^ Criteria as published by CLSI (2025), EUCAST (2025), and US FDA (2025).

^*b*^ CLSI breakpoints applied, since recognized by the US FDA.

^*c*^ Intermediate results can be considered susceptible if increased antibiotic exposures can be achieved.

^*d*^ Using non-meningitis breakpoints.

^*e*^ DTR, phenotype defined as nonsusceptible MIC results (CLSI) to piperacillin-tazobactam, ceftazidime, cefepime, aztreonam, meropenem, imipenem, ciprofloxacin, and levofloxacin.

Among DTR isolates nonsusceptible to ≥1 or more of the three recommended BL-BLI combinations, cefiderocol (82.0%–97.5%) maintained consistent MIC_50_ and MIC_90_ values of 0.25 mg/L and 2 mg/L, respectively (Table 1). In general, this consistency of MIC_50_ and MIC_90_ values for cefiderocol was also observed among DTR subsets from US and EUR hospitals (Tables S2 and S3). Low susceptibilities (≤30%) were observed for the three recommended BL-BLI agents when non-susceptibility was observed for any of these combination agents (Table 1). Cefiderocol also maintained good activity against isolates that were nonsusceptible to 2–3 of the BL-BLI combinations (79.3%–96.3%), whereas susceptibilities of the BL-BLI combinations ranged from 0.0% to 18.5% (Table 1).

Carbapenemase genes were detected in 19.6% (74/377) of DTR isolates, with class B MBL-type genes being the most common genes, and identified in 93.2% (69/74) of carbapenemase carriers, while genes encoding class A serine carbapenemases were identified in 6.8% (5/74). A single isolate from Greece possessed genes encoding VIM-2 and GES-5 (Table 3). These carbepenemase-carrying isolates originated from the USA (*N* = 6), 12 EUR countries (*N* = 61), Israel (*N* = 1), and Turkey (*N* = 6), and most of the isolates from EUR were collected from Italian (20.6%; 14/68) and Greek (22.1%; 15/68) sites (data not shown). Cefiderocol had MIC_50_ and MIC_90_ values of 0.25 mg/L and 2 mg/L, respectively, against carbapenemase-carrying DTR isolates, whereas MIC_50_ and MIC_90_ values of 0.12 mg/L and 1 mg/L, respectively, against non-carbapenemase DTR *P. aeruginosa* (Table 3). In addition, 82.6% to 100% of non-carbapenemase and carbapenemase-carrying isolates and respective subsets were susceptible to cefiderocol. In contrast, the IDSA recommended BL-BLI combinations were inactive against the carbapenemase-carrying subsets and showed limited activity against the non-carbapenemase group (63.7%–69.6% susceptible) (Table 3).

**TABLE 3:** Activity of cefiderocol and comparator BL-BLI combinations against DTR *P. aeruginosa* isolates collected in US and European medical centers (2020–2023)

		% susceptible^*b*^					
	FDC^*a*^				CAZ-AVI^*a*^	IMI-REL^*a*^	TOL-TAZ^*a*^
Genotype^*a*^ (*n*; %)	MIC_50/90_	CLSI	EUCAST	FDA		CLSI	
Carbapenemase^*c*^ (74; 19.6)	0.25/2	97.3	94.6	83.8	5.4	0.0	0.0
Carbapenemase +NS to BI-BLI^*d*^ (70; 18.6)	0.25/2	97.1	94.3	82.9	0.0	0.0	0.0
MBL (69; 93.2)	0.25/2	97.1	94.2	82.6	0.0	0.0	0.0
VIM (53; 71.6)	0.25/2	100	98.1	88.7	0.0	0.0	0.0
Non-carbapenemase^*e*^ (303; 80.4)	0.12/1	98.3	97.4	90.1	63.7	69.6	67.7

^*a*^ FDC, cefiderocol; CAZ-AVI, ceftazidime-avibactam; IMI-REL, imipenem-relebactam; TOL-TAZ, ceftolozane-tazobactam; NS, nonsusceptible. MBL, metallo-β-lactamase; VIM, Verona integron-mediated metallo-β-lactamase.

^*b*^ CLSI (2025), EUCAST (2025) and the FDA breakpoints for cefiderocol applied. CLSI (2025) breakpoints applied for comparator agents.

^*c*^ Includes isolates with *bla*_GES-4 _(1), *bla*_GES-5 _(1), *bla*_GES-6 _(3), *bla*_IMP-1 _(2), *bla*_IMP-7 _(5), *bla*_IMP-8 _(1), *bla*_IMP-13 _(1), *bla*_NDM-1 _(7), *bla*_VIM-1 _(6), *bla*_VIM-1_ + *bla*_HMB-1 _(1), *bla*_VIM-2 _(41), *bla*_VIM-2_ + *bla*_GES-5_ (1)_, _*bla*_VIM-4 _(3), and *bla*_VIM-5 _(1).

^*d*^ Includes isolates nonsusceptible (CLSI) to the three recommended BL-BLI combinations and carrying acquired carbapenemases, as follows: *bla*_GES-4 _(1), *bla*_IMP-1 _(2), *bla*_IMP-7 _(5), *bla*_IMP-8 _(1), *bla*_IMP-13 _(1), *bla*_NDM-1 _(7), *bla*_VIM-1 _(6), *bla*_VIM-1_ + *bla*_HMB-1 _(1), *bla*_VIM-2 _(41), *bla*_VIM-2_ + *bla*_GES-5_ (1)_, _*bla*_VIM-4 _(3), and *bla*_VIM-5 _(1).

^*e*^ Includes isolates where acquired carbapenemases were not detected.

**Fig 1:**
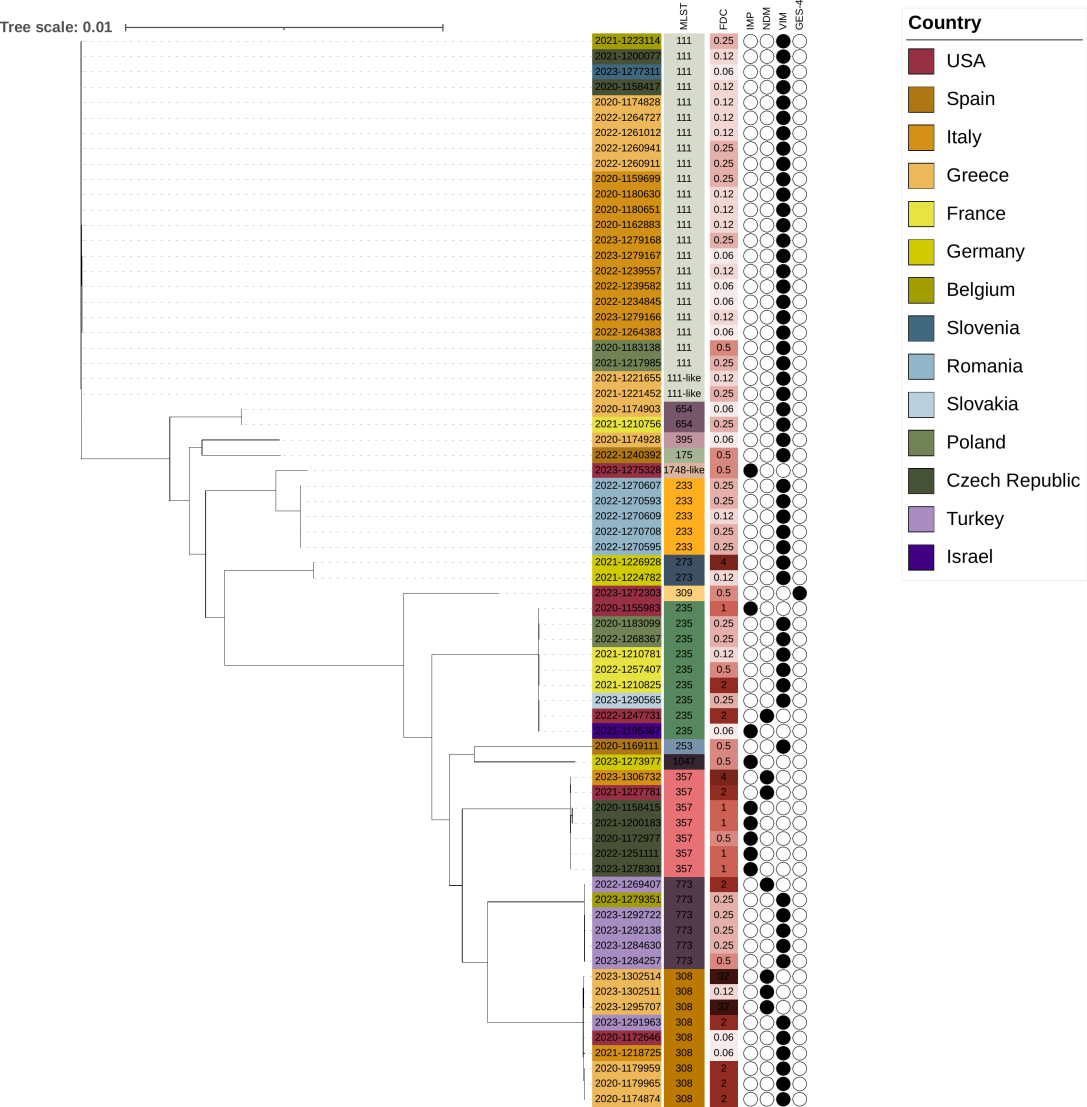
The phylogenetic tree of 70 DTR *P. aeruginosa* nonsusceptible (CLSI) to the three recommended BL-BLI combinations and carrying carbapenemases was constructed by creating a core genome MLST (cgMLST; 3,867 loci) for each isolate using chewBBACA v3.2.0. and based on the scheme for *P. aeruginosa* available at cgmlst.org. A multiple genome alignment was performed using MUSCLE v3.8.11 and an unrooted maximum likelihood tree was created with FastTree v2.2.11. The tree and associated metadata was curated with iTol v6.8.2. Isolates are identified with the year of their isolation and a unique collection number. MLST and cefiderocol (FDC) MIC results are color-coded, with the MIC displayed on a sliding color scale from the lowest (lightest shade) to the highest values (darkest shade). The presence of carbapenemase genes is denoted for each isolate as a filled circle.

Among the DTR isolates carrying carbapenemases, 94.6% (70/74) were nonsusceptible to all three recommended BL-BLI agents, and cefiderocol (82.9%–97.1%) remained active against these isolates. The phylogeny of these 70 DTR isolates is shown in Fig. 1. The majority of these DTR isolates carried *bla*_VIM_ (75.7%), followed by similar proportions of *bla*_IMP_ (12.9%) and *bla*_NDM_ (10%). One isolate carried *bla*_GES-4_ (Table 3). These isolates were collected in the USA (*n* = 6; 8.6%), Eastern Europe (*n* = 40; 57.1%), and Western Europe (*n* = 24; 34.3%). Overall, this subset of isolates was comprised of 14 STs, with 6 isolates coming from singleton STs. Most isolates belonged to ST111 (34.3%; 24/70), followed by equal numbers of ST235 and ST308 (12.9%; 9/70). All but 2 isolates were susceptible to cefiderocol and inhibited at MIC of ≤4 mg/L (CLSI breakpoint). Among the 12 isolates showing cefiderocol MIC of 2–32 mg/L, 6 carried *bla*_VIM_ and 6 carried *bla*_NDM_. All but 1 isolate (MIC, 0.12 mg/L) carrying *bla*_NDM_ had elevated cefiderocol MIC (2–32 mg/L). The two isolates with a cefiderocol MIC of 32 mg/L belonged to ST 308 and were recovered from a single site in Greece.

## DISCUSSION

This study reports the activity of cefiderocol and comparator agents against contemporary *P. aeruginosa* clinical isolates collected from US and EUR medical centers, but most specifically against those with a DTR phenotype. The results presented here mirror those of previous studies (22, 45) and demonstrated the activity of cefiderocol (98.6%–99.8% susceptible) against the general population of *P. aeruginosa,* and the three IDSA-recommended BL-BLI agents TOL-TAZ, CAZ-AVI, and IMI-REL (95.9%–96.5% susceptible). However, cefiderocol (88.9%–98.1% susceptible) remained active against the DTR group, whereas the three BL-BLI combinations (52.3%–56.0% susceptible) were less active. Even when tested against a subset nonsusceptible to these three BL-BLI combinations and carrying carbapenemases, cefiderocol inhibited 97.1% of isolates at the CLSI breakpoint for susceptibility (≤4 mg/L).

The results presented here are relevant because IDSA currently recommends the use of TOL-TAZ, CAZ-AVI, IMI-REL, or cefiderocol for DTR infections caused by *P. aeruginosa* (16). However, these results demonstrate cefiderocol may rather be given first consideration for treating infections caused by DTR *P. aeruginosa* because approximately 63% of these isolates were nonsusceptible to at least one BL-BLI, as well as a high degree of cross- resistance to these combinations (Table 1). These results corroborate previous reports describing the development of cross resistance to these combinations following exposure *in vitro*, in clinical settings, and in surveillance studies (46–49). Furthermore, cross-resistance among BL-BLI combinations was observed elsewhere (33, 50–52), suggesting early use of cefiderocol may be more appropriate.

Approximately 80% of DTR isolates lacked acquired carbapenemases, and those that did acquire carbapenemases originated from EUR countries (91.9%). These data emphasize the importance of non-acquired resistance mechanisms among *P. aeruginosa* from the USA (30–32). The nonsusceptible phenotype observed for the BL-BLI combinations may be simply due to the presence of multiple resistance mechanisms. But, the nonsusceptible phenotypes to these combinations seem to get exacerbated by the presence of carbapenemases (53–56), which were more prevalent within the EUR region, especially in Eastern Europe (48, 55). Isolates expressing carbapenemases were diverse with 14 STs encountered and isolated in 15 different countries, but a majority of these isolates expressed metallo-β-lactamases. Aztreonam-avibactam, a BL-BLI combination with good activity against metallo-β-lactamase-carrying Enterobacterales, was ineffective against these metallo-β-lactamase-carrying *P. aeruginosa*.

In contrast to the elevated nonsusceptibility and high degree of cross-resistance observed among the three IDSA-recommended BL-BLI combinations, a smaller percentage of isolates was nonsusceptible to cefiderocol. This indicates the lack, or reduced presence, of overlapping mechanisms conferring resistance to cefiderocol and these BL-BLI agents. This may be explained by the fact that cefiderocol enters the cell through specific siderophore transport proteins, directly linking the uptake of this molecule to cellular iron homeostasis, counteracting the common resistance mechanisms linked to other antibiotics in *P. aeruginosa* (20, 57, 58). Although previously described, resistance mechanisms to cefiderocol appear to be uncommon among the surveillance isolates included in this study based on the MIC results observed; however, this study did not systematically investigate or present data on these isolates nonsusceptible to cefiderocol or β-lactam resistance mechanisms other than the presence of β-lactamase genes (57, 59, 60).

### Conclusions

Overall, cefiderocol demonstrated high activity against a large collection of *P. aeruginosa*, including DTR isolates. In contrast, the three currently recommended BL-BLI combinations for treating certain *P. aeruginosa* infection types according to IDSA guidelines demonstrated limited activity, especially against isolates carrying carbapenemase genes, and high degree of cross-resistance. Cefiderocol also sustained activity against a diverse array of isolates with various BL-BLI nonsusceptible phenotypes, and regardless of carbapenemase status. The findings reported here support the use of cefiderocol as a first treatment option for infections caused by DTR *P. aeruginosa*, where treatment with the current IDSA-recommended BL-BLI combinations may be refractory.

## SUPPLEMENTAL MATERIAL

Supplemental material spectrum.02079-25-s0001.docx Tables S1 to S3.

ASM does not own the copyrights to Supplemental Material that may be linked to, or accessed through, an article. The authors have granted ASM a non-exclusive, world-wide license to publish the Supplemental Material files. Please contact the corresponding author directly for reuse.

## References

[1] Canton R, Doi Y, Simner PJ. 2022. Treatment of carbapenem-resistant Pseudomonas aeruginosa infections: a case for cefiderocol. Expert Rev Anti Infect Ther 20:1077–1094. doi:10.1080/14787210.2022.2071701 35502603

[2] Berrazeg M, Jeannot K, Ntsogo Enguéné VY, Broutin I, Loeffert S, Fournier D, Plésiat P. 2015. Mutations in β-lactamase AmpC increase resistance of Pseudomonas aeruginosa isolates to antipseudomonal cephalosporins. Antimicrob Agents Chemother 59:6248–6255. doi:10.1128/AAC.00825-15 26248364 PMC4576058

[3] Cabot G, Kim K, Mark BL, Oliver A, Khajehpour M. 2023. Biochemical insights into imipenem collateral susceptibility driven by ampC mutations conferring ceftolozane/tazobactam resistance in Pseudomonas aeruginosa. Antimicrob Agents Chemother 67:e0140922. doi:10.1128/aac.01409-22 36715512 PMC9933714

[4] Barceló IM, Jordana-Lluch E, Escobar-Salom M, Torrens G, Fraile-Ribot PA, Cabot G, Mulet X, Zamorano L, Juan C, Oliver A. 2022. Role of enzymatic activity in the biological cost associated with the production of AmpC β-lactamases in Pseudomonas aeruginosa. Microbiol Spectr 10:e0270022. doi:10.1128/spectrum.02700-22 36214681 PMC9604156

[5] Torrens G, Hernandez SB, Ayala JA, Moya B, Juan C, Cava F, Oliver A. 2019. Different signaling. mSystems 4:e00524-19. doi:10.1128/msystems.00524-19 31796566 PMC6890930

[6] Castanheira M, Doyle TB, Smith CJ, Mendes RE, Sader HS. 2019. Combination of MexAB-OprM overexpression and mutations in efflux regulators, PBPs and chaperone proteins is responsible for ceftazidime/avibactam resistance in Pseudomonas aeruginosa clinical isolates from US hospitals. J Antimicrob Chemother 74:2588–2595. doi:10.1093/jac/dkz243 31225882

[7] Nakae T, Nakajima A, Ono T, Saito K, Yoneyama H. 1999. Resistance to β-lactam antibiotics in Pseudomonas aeruginosa due to interplay between the MexAB-OprM efflux pump and β-lactamase. Antimicrob Agents Chemother 43:1301–1303. doi:10.1128/AAC.43.5.1301 10223959 PMC89266

[8] Rodríguez-Martínez J-M, Poirel L, Nordmann P. 2009. Molecular epidemiology and mechanisms of carbapenem resistance in Pseudomonas aeruginosa. Antimicrob Agents Chemother 53:4783–4788. doi:10.1128/AAC.00574-09 19738025 PMC2772299

[9] Horcajada JP, Montero M, Oliver A, Sorlí L, Luque S, Gómez-Zorrilla S, Benito N, Grau S. 2019. Epidemiology and treatment of multidrug-resistant and extensively drug-resistant Pseudomonas aeruginosa infections. Clin Microbiol Rev 32:e00031-19. doi:10.1128/CMR.00031-19 31462403 PMC6730496

[10] Kao C-Y, Chen S-S, Hung K-H, Wu H-M, Hsueh P-R, Yan J-J, Wu J-J. 2016. Overproduction of active efflux pump and variations of OprD dominate in imipenem-resistant Pseudomonas aeruginosa isolated from patients with bloodstream infections in Taiwan. BMC Microbiol 16:107. doi:10.1186/s12866-016-0719-2 27296461 PMC4906909

[11] Castanheira M, Doyle TB, Hubler CM, Collingsworth TD, DeVries S, Mendes RE. 2022. The plethora of resistance mechanisms in Pseudomonas aeruginosa: transcriptome analysis reveals a potential role of lipopolysaccharide pathway proteins to novel β-lactam/β-lactamase inhibitor combinations. J Glob Antimicrob Resist 31:72–79. doi:10.1016/j.jgar.2022.07.021 35931381

[12] Kocsis B, Gulyás D, Szabó D. 2021. Diversity and distribution of resistance markers in Pseudomonas aeruginosa international high-risk clones. Microorganisms 9:359. doi:10.3390/microorganisms9020359 33673029 PMC7918723

[13] Hernández-García M, García-Castillo M, Nieto-Torres M, Bou G, Ocampo-Sosa A, Pitart C, Gracia-Ahufinger I, Mulet X, Pascual Á, Tormo N, Oliver A, Ruiz-Garbajosa P, Cantón R. 2024. Deciphering mechanisms affecting cefepime-taniborbactam in vitro activity in carbapenemase-producing Enterobacterales and carbapenem-resistant Pseudomonas spp. isolates recovered during a surveillance study in Spain. Eur J Clin Microbiol Infect Dis 43:279–296. doi:10.1007/s10096-023-04697-4 38041722

[14] Doumith M, Alhassinah S, Alswaji A, Alzayer M, Alrashidi E, Okdah L, Aljohani S, Group NAS, Balkhy HH, Alghoribi MF. 2021. Genomic characterization of carbapenem-non-susceptible Pseudomonas aeruginosa clinical isolates from Saudi Arabia revealed a global dissemination of GES-5-producing ST235 and VIM-2-producing ST233 sub-lineages. Front Microbiol 12:765113. doi:10.3389/fmicb.2021.765113 35069471 PMC8770977

[15] Gomis-Font MA, Cabot G, López-Argüello S, Zamorano L, Juan C, Moyá B, Oliver A. 2022. Comparative analysis of in vitro dynamics and mechanisms of ceftolozane/tazobactam and imipenem/relebactam resistance development in Pseudomonas aeruginosa XDR high-risk clones. J Antimicrob Chemother 77:957–968. doi:10.1093/jac/dkab496 35084040

[16] Tamma PD, Heil EL, Justo JA, Mathers AJ, Satlin MJ, Bonomo RA. 2024. Infectious diseases society of America 2024 guidance on the treatment of antimicrobial-resistant gram-negative infections. Clin Infect Dis:ciae403. doi:10.1093/cid/ciae403doi:39108079

[17] Del Barrio-Tofiño E, López-Causapé C, Oliver A. 2020. Pseudomonas aeruginosa epidemic high-risk clones and their association with horizontally-acquired β-lactamases: 2020 update. Int J Antimicrob Agents 56:106196. doi:10.1016/j.ijantimicag.2020.106196 33045347

[18] Cosentino F, Viale P, Giannella M. 2023. MDR/XDR/PDR or DTR? Which definition best fits the resistance profile of Pseudomonas aeruginosa? Curr Opin Infect Dis 36:564–571. doi:10.1097/QCO.0000000000000966 37930070 PMC10836784

[19] Ito A, Sato T, Ota M, Takemura M, Nishikawa T, Toba S, Kohira N, Miyagawa S, Ishibashi N, Matsumoto S, Nakamura R, Tsuji M, Yamano Y. 2018. In vitro antibacterial properties of cefiderocol, a novel siderophore cephalosporin, against Gram-negative bacteria. Antimicrob Agents Chemother 62:e01454-17. doi:10.1128/AAC.01454-17 PMC5740388 29061741

[20] Ito A, Nishikawa T, Matsumoto S, Yoshizawa H, Sato T, Nakamura R, Tsuji M, Yamano Y. 2016. Siderophore cephalosporin cefiderocol utilizes ferric iron transporter systems for antibacterial activity against Pseudomonas aeruginosa. Antimicrob Agents Chemother 60:7396–7401. doi:10.1128/AAC.01405-16 27736756 PMC5119021

[21] Longshaw C, Manissero D, Tsuji M, Echols R, Yamano Y. 2020. In vitro activity of the siderophore cephalosporin, cefiderocol, against molecularly characterized, carbapenem-non-susceptible gram-negative bacteria from Europe. JAC Antimicrob Resist 2:dlaa060. doi:10.1093/jacamr/dlaa060 34223017 PMC8210120

[22] Shortridge D, Streit JM, Mendes R, Castanheira M. 2022. In vitro activity of cefiderocol against U.S. and European gram-negative clinical isolates collected in 2020 as part of the SENTRY antimicrobial surveillance program. Microbiol Spectr 10:e0271221. doi:10.1128/spectrum.02712-21 35262394 PMC9045385

[23] Lasarte-Monterrubio C, Fraile-Ribot PA, Vázquez-Ucha JC, Cabot G, Guijarro-Sánchez P, Alonso-García I, Rumbo-Feal S, Galán-Sánchez F, Beceiro A, Arca-Suárez J, Oliver A, Bou G. 2022. Activity of cefiderocol, imipenem/relebactam, cefepime/taniborbactam and cefepime/zidebactam against ceftolozane/tazobactam- and ceftazidime/avibactam-resistant Pseudomonas aeruginosa. J Antimicrob Chemother 77:2809–2815. doi:10.1093/jac/dkac241 35904000

[24] Ito A, Nishikawa T, Ota M, Ito-Horiyama T, Ishibashi N, Sato T, Tsuji M, Yamano Y. 2018. Stability and low induction propensity of cefiderocol against chromosomal AmpC β-lactamases of Pseudomonas aeruginosa and Enterobacter cloacae. J Antimicrob Chemother 73:3049–3052. doi:10.1093/jac/dky317 30188999 PMC6198743

[25] Schellong P, Wennek-Klose J, Spiegel C, Rödel J, Hagel S. 2024. Successful outpatient parenteral antibiotic therapy with cefiderocol for osteomyelitis caused by multi-drug resistant gram-negative bacteria: a case report. JAC Antimicrob Resist 6:dlae015. doi:10.1093/jacamr/dlae015 38328266 PMC10848891

[26] El Ghali A, Kunz Coyne AJ, Lucas K, Tieman M, Xhemali X, Lau S-P, Iturralde G, Purdy A, Holger DJ, Garcia E, Veve MP, Rybak MJ. 2024. Cefiderocol: early clinical experience for multi-drug resistant gram-negative infections. Microbiol Spectr 12:e0310823. doi:10.1128/spectrum.03108-23doi:38206034 PMC10846278

[27] Piccica M, Spinicci M, Botta A, Bianco V, Lagi F, Graziani L, Faragona A, Parrella R, Giani T, Bartolini A, Morroni G, Bernardo M, Rossolini GM, Tavio M, Giacometti A, Bartoloni A. 2023. Cefiderocol use for the treatment of infections by carbapenem-resistant gram-negative bacteria: an Italian multicentre real-life experience. J Antimicrob Chemother 78:2752–2761. doi:10.1093/jac/dkad298 37807834 PMC10631827

[28] Gill CM, Aktaþ E, Alfouzan W, Bourassa L, Brink A, Burnham C-AD, Canton R, Carmeli Y, Falcone M, Kiffer C, Marchese A, Martinez O, Pournaras S, Satlin M, Seifert H, Thabit AK, Thomson KS, Villegas MV, Nicolau DP, ERACE-PA Global Study Group. 2021. The ERACE-PA global surveillance program: ceftolozane/tazobactam and ceftazidime/avibactam in vitro activity against a global collection of carbapenem-resistant Pseudomonas aeruginosa. Eur J Clin Microbiol Infect Dis 40:2533–2541. doi:10.1007/s10096-021-04308-0 34291323 PMC8590662

[29] Karlowsky JA, Lob SH, Estabrook MA, Siddiqui F, DeRyke CA, Young K, Motyl MR, Sahm DF. 2023. Susceptibility profile and β-lactamase content of global Pseudomonas aeruginosa isolates resistant to ceftolozane/tazobactam and/or imipenem/relebactam-SMART 2016-21. JAC Antimicrob Resist 5:dlad080. doi:10.1093/jacamr/dlad080 37388237 PMC10306085

[30] Shields RK, Stellfox ME, Kline EG, Samanta P, Van Tyne D. 2022. Evolution of imipenem-relebactam resistance following treatment of multidrug-resistant Pseudomonas aeruginosa pneumonia. Clin Infect Dis 75:710–714. doi:10.1093/cid/ciac097 35136967 PMC9890448

[31] Gomis-Font MA, Cabot G, Sánchez-Diener I, Fraile-Ribot PA, Juan C, Moya B, Zamorano L, Oliver A. 2020. In vitro dynamics and mechanisms of resistance development to imipenem and imipenem/relebactam in Pseudomonas aeruginosa. J Antimicrob Chemother 75:2508–2515. doi:10.1093/jac/dkaa206 32514525

[32] Castanheira M, Mills JC, Farrell DJ, Jones RN. 2014. Mutation-driven β-lactam resistance mechanisms among contemporary ceftazidime-nonsusceptible Pseudomonas aeruginosa isolates from U.S. hospitals. Antimicrob Agents Chemother 58:6844–6850. doi:10.1128/AAC.03681-14 25182652 PMC4249397

[33] Fraile-Ribot PA, Cabot G, Mulet X, Periañez L, Martín-Pena ML, Juan C, Pérez JL, Oliver A. 2018. Mechanisms leading to in vivo ceftolozane/tazobactam resistance development during the treatment of infections caused by MDR Pseudomonas aeruginosa. J Antimicrob Chemother 73:658–663. doi:10.1093/jac/dkx424 29149337

[34] Cabot G, Bruchmann S, Mulet X, Zamorano L, Moyà B, Juan C, Haussler S, Oliver A. 2014. Pseudomonas aeruginosa ceftolozane-tazobactam resistance development requires multiple mutations leading to overexpression and structural modification of AmpC. Antimicrob Agents Chemother 58:3091–3099. doi:10.1128/AAC.02462-13 24637685 PMC4068469

[35] CLSI. 2024. M07Ed12. Methods for dilution antimicrobial susceptibility tests for bacteria that grow aerobically. CLSI, Berwyn, PA.

[36] CLSI. 2025. M100Ed35. Performance standards for antimicrobial susceptibility testing: 35th informational supplement. CLSI, Wayne, PA.

[37] Breakpoint tables for interpretation of MICs and zone diameters. 2025. Available from: http://www.eucast.org/fileadmin/src/media/PDFs/EUCAST_files/Breakpoint_tables/v_15.0_Breakpoint_Tables.pdf. Retrieved 1 Apr 2025.

[38] US Food and Drug Administration. 2025. Antimicrobial Susceptibility Test Interpretive Criteria (STIC). Available from: https://www.fda.gov/drugs/development-resources/antibacterial-susceptibility-test-interpretive-criteria. Retrieved 30 Sep 2025.

[39] Bankevich A, Nurk S, Antipov D, Gurevich AA, Dvorkin M, Kulikov AS, Lesin VM, Nikolenko SI, Pham S, Prjibelski AD, Pyshkin AV, Sirotkin AV, Vyahhi N, Tesler G, Alekseyev MA, Pevzner PA. 2012. SPAdes: a new genome assembly algorithm and its applications to single-cell sequencing. J Comput Biol 19:455–477. doi:10.1089/cmb.2012.0021 22506599 PMC3342519

[40] Silva M, Machado MP, Silva DN, Rossi M, Moran-Gilad J, Santos S, Ramirez M, Carriço JA. 2018. chewBBACA: A complete suite for gene-by-gene schema creation and strain identification. Microb Genom 4:e000166. doi:10.1099/mgen.0.000166 29543149 PMC5885018

[41] Tönnies H, Prior K, Harmsen D, Mellmann A. 2021. Establishment and evaluation of a core genome multilocus sequence typing scheme for whole-genome sequence-based typing of Pseudomonas aeruginosa. J Clin Microbiol 59:e01987-20. doi:10.1128/JCM.01987-20 33328175 PMC8106710

[42] Edgar RC. 2004. MUSCLE: multiple sequence alignment with high accuracy and high throughput. Nucleic Acids Res 32:1792–1797. doi:10.1093/nar/gkh340 15034147 PMC390337

[43] Price MN, Dehal PS, Arkin AP. 2010. FastTree 2 – approximately maximum-likelihood trees for large alignments. PLoS One 5:e9490. doi:10.1371/journal.pone.0009490 20224823 PMC2835736

[44] Letunic I, Bork P. 2021. Interactive tree of life (iTOL) v5: an online tool for phylogenetic tree display and annotation. Nucleic Acids Res 49:W293–W296. doi:10.1093/nar/gkab301 33885785 PMC8265157

[45] Santerre Henriksen A, Jeannot K, Oliver A, Perry JD, Pletz MW, Stefani S, Morrissey I, Longshaw C, Investigators AS. 2024. In vitro activity of cefiderocol against European Pseudomonas aeruginosa and Acinetobacter spp., including isolates resistant to meropenem and recent β-lactam/β-lactamase inhibitor combinations. Microbiol Spectr 12:e0383623. doi:10.1128/spectrum.03836-23 38483164 PMC10986614

[46] Shields RK, Kline EG, Squires KM, Van Tyne D, Doi Y. 2023. In vitro activity of cefiderocol against Pseudomonas aeruginosa demonstrating evolved resistance to novel β-lactam/β-lactamase inhibitors. JAC Antimicrob Resist 5:dlad107. doi:10.1093/jacamr/dlad107 37795425 PMC10546814

[47] Sader HS, Mendes RE, Arends SJR, Carvalhaes CG, Shortridge D, Castanheira M. 2023. Comparative activity of newer β-lactam/β-lactamase inhibitor combinations against Pseudomonas aeruginosa isolates from US medical centres (2020-2021). Int J Antimicrob Agents 61:106744. doi:10.1016/j.ijantimicag.2023.106744 36738849

[48] Gill CM, Nicolau DP. 2022. Phenotypic and genotypic profile of ceftolozane/tazobactam-non-susceptible, carbapenem-resistant Pseudomonas aeruginosa. J Antimicrob Chemother 78:252–256. doi:10.1093/jac/dkac385 36411249 PMC9780534

[49] Castanheira M, Kimbrough JH, Lindley J, Doyle TB, Ewald JM, Sader HS. 2024. In vitro development of resistance against antipseudomonal agents: comparison of novel β-lactam/β-lactamase inhibitor combinations and other β-lactam agents. Antimicrob Agents Chemother 68:e0136323. doi:10.1128/aac.01363-23 38526050 PMC11064483

[50] Ruedas-López A, Alonso-García I, Lasarte-Monterrubio C, Guijarro-Sánchez P, Gato E, Vázquez-Ucha JC, Vallejo JA, Fraile-Ribot PA, Fernández-Pérez B, Velasco D, Gutiérrez-Urbón JM, Oviaño M, Beceiro A, González-Bello C, Oliver A, Arca-Suárez J, Bou G. 2022. Selection of ampC β-lactamase variants and metallo-β-Lactamases leading to ceftolozane/tazobactam and ceftazidime/avibactam resistance during treatment of MDR/XDR Pseudomonas aeruginosa infections. Antimicrob Agents Chemother 66:e0206721. doi:10.1128/AAC.02067-21 34930034 PMC8846482

[51] Boulant T, Jousset AB, Bonnin RA, Barrail-Tran A, Borgel A, Oueslati S, Naas T, Dortet L. 2019. A 2.5-years within-patient evolution of a Pseudomonas aeruginosa with in vivo acquisition of ceftolozane-tazobactam and ceftazidime-avibactam resistance upon treatment. Antimicrob Agents Chemother 63:e01637-19. doi:10.1128/AAC.01637-19 31636072 PMC6879234

[52] Poirel L, Ortiz de la Rosa JM, Sadek M, Nordmann P. 2022. Impact of acquired broad-spectrum β-lactamases on susceptibility to cefiderocol and newly developed β-lactam/β-lactamase inhibitor combinations in Escherichia coli and Pseudomonas aeruginosa. Antimicrob Agents Chemother 66:e0003922. doi:10.1128/aac.00039-22 35315685 PMC9017383

[53] Adámková V, Mareković I, Szabó J, Pojnar L, Billová S, Horvat Herceg S, Kuraieva A, Możejko-Pastewka B. 2022. Antimicrobial activity of ceftazidime-avibactam and comparators against Pseudomonas aeruginosa and Enterobacterales collected in Croatia, Czech Republic, Hungary, Poland, Latvia and Lithuania: ATLAS surveillance program, 2019. Eur J Clin Microbiol Infect Dis 41:989–996. doi:10.1007/s10096-022-04452-1 35596097 PMC9135846

[54] Reyes J, Komarow L, Chen L, Ge L, Hanson BM, Cober E, Herc E, Alenazi T, Kaye KS, Garcia-Diaz J, et al.. 2023. Global epidemiology and clinical outcomes of carbapenem-resistant Pseudomonas aeruginosa and associated carbapenemases (POP): a prospective cohort study. The Lancet Microbe 4:e159–e170. doi:10.1016/S2666-5247(22)00329-9 36774938 PMC10016089

[55] Karlowsky JA, Lob SH, Siddiqui F, Akrich B, DeRyke CA, Young K, Motyl MR, Hawser SP, Sahm DF. 2023. In vitro activity of ceftolozane/tazobactam against multidrug-resistant Pseudomonas aeruginosa from patients in Western Europe: SMART 2017-2020. Int J Antimicrob Agents 61:106772. doi:10.1016/j.ijantimicag.2023.106772 36878411

[56] Kazmierczak KM, Rabine S, Hackel M, McLaughlin RE, Biedenbach DJ, Bouchillon SK, Sahm DF, Bradford PA. 2016. Multiyear, multinational survey of the incidence and global distribution of metallo-β-lactamase-producing Enterobacteriaceae and Pseudomonas aeruginosa. Antimicrob Agents Chemother 60:1067–1078. doi:10.1128/AAC.02379-15 26643349 PMC4750703

[57] Luscher A, Moynié L, Auguste PS, Bumann D, Mazza L, Pletzer D, Naismith JH, Köhler T. 2018. TonB-dependent receptor repertoire of Pseudomonas aeruginosa for uptake of siderophore-drug conjugates. Antimicrob Agents Chemother 62:e00097-18. doi:10.1128/AAC.00097-18 29555629 PMC5971595

[58] Moynié L, Luscher A, Rolo D, Pletzer D, Tortajada A, Weingart H, Braun Y, Page MGP, Naismith JH, Köhler T. 2017. Structure and function of the PiuA and PirA siderophore-drug receptors from Pseudomonas aeruginosa and Acinetobacter baumannii. Antimicrob Agents Chemother 61:e02531-16. doi:10.1128/AAC.02531-16 28137795 PMC5365723

[59] McPherson CJ, Aschenbrenner LM, Lacey BM, Fahnoe KC, Lemmon MM, Finegan SM, Tadakamalla B, O’Donnell JP, Mueller JP, Tomaras AP. 2012. Clinically relevant gram-negative resistance mechanisms have no effect on the efficacy of MC-1, a novel siderophore-conjugated monocarbam. Antimicrob Agents Chemother 56:6334–6342. doi:10.1128/AAC.01345-12 23027195 PMC3497185

[60] Luscher A, Gasser V, Bumann D, Mislin GLA, Schalk IJ, Köhler T. 2022. Plant-derived catechols are substrates of tonB-dependent transporters and sensitize Pseudomonas aeruginosa to siderophore-drug conjugates. mBio 13:e0149822. doi:10.1128/mbio.01498-22 35770947 PMC9426570

