# Supplementary material for: Activity of cefiderocol against Pseudomonas aeruginosa from the USA and Europe (2020–2023) with difficult-to-treat resistance phenotype, including those nonsusceptible to recently developed β-lactam/β-lactamase inhibitor combinations: results from the SENTRY antimicrobial surveillance program
Source: Microbiol Spectr. 2025 Oct 13;13(11):e02079-25. doi: 10.1128/spectrum.02079-25 (PMC12584632; doi:10.1128/spectrum.02079-25)
Supplement: Supplemental material — Tables S1 to S3. [file spectrum.02079-25-s0001.docx]

Table S1. Geographic and infection type distributions of all *P. aeruginosa* and the DTR subset collected in US and European medical centers (2020-2023).

| Continent/Region/Country | Year | | | | Total (DTR) |
| --- | --- | --- | --- | --- | --- |
|  | 2020 | 2021 | 2022 | 2023 |  |
| Europe | **1,213** | **1,227** | **1,486** | **1246** | **5172 (223)** |
| Eastern | ***347*** | ***353*** | ***469*** | ***402*** | ***1571 (119)*** |
| Czech Republic | 32 | 10 | 23 | 28 | 93 (8) |
| Greece | 47 | 45 | 51 | 30 | 173 (23) |
| Hungary | 30 | 36 | 25 | 64 | 155 (2) |
| Israel | 38 | 94 | 129 | 106 | 367 (13) |
| Poland | 75 | 36 | 49 | 19 | 179 (18) |
| Romania | 17 | 10 | 71 | 13 | 111 (13) |
| Slovakia | - | - | - | 19 | 19 (5) |
| Slovenia | 40 | 35 | 41 | 50 | 166 (6) |
| Turkey | 68 | 87 | 80 | 73 | 308 (31) |
| Western | ***866*** | ***874*** | ***1017*** | ***844*** | ***3601 (104)*** |
| Belgium | 29 | 22 | 26 | 22 | 99 (9) |
| France | 90 | 160 | 164 | 118 | 532 (15) |
| Germany | 180 | 142 | 168 | 164 | 654 (20) |
| Ireland | 24 | 24 | 33 | 39 | 120 (8) |
| Italy | 196 | 218 | 205 | 171 | 790 (21) |
| Portugal | 27 | 42 | 38 | 43 | 150 (5) |
| Spain | 190 | 173 | 198 | 139 | 700 (21) |
| Sweden | 37 | 22 | 30 | 44 | 133 (3) |
| Switzerland | 34 | 34 | 50 | 36 | 154 (0) |
| UK | 59 | 37 | 105 | 68 | 269 (2) |
| North America (USA) | **1069** | **1172** | **1143** | **1016** | **4400** **(154)** |
| Total | **2282** | **2399** | **2629** | **2262** | **9572** **(377)** |
| Infection Type |  |  |  |  |  |
| Pneumonia in Hospitalized Patients | 1153 | 1271 | 1385 | 1385 | 5061 (222) |
| Skin and Soft Tissue Infection | 465 | 444 | 543 | 376 | 1828 (69) |
| Bloodstream Infection | 335 | 356 | 360 | 347 | 1398 (40) |
| Urinary Tract Infection | 222 | 222 | 223 | 201 | 868 (33) |
| Intra-abdominal Infection | 106 | 105 | 117 | 64 | 392 (12) |
| Other Infection Types | 1 | 1 | 1 | 22 | 25 (1) |

Table S2. Activity of cefiderocol and comparator BL-BLI combinations against DTR *P. aeruginosa* collected in US medical centers (2020-2023)

|  |  |  | % susceptible^a^ | | | | | | |
| --- | --- | --- | --- | --- | --- | --- | --- | --- | --- |
|  |  |  |  | FDC |  |  | CAZ-AVI | IMI-REL | TOL-TAZ |
| Susceptibility Profile (n; %) | MIC_50/90_ |  | CLSI | EUCAST | FDA |  | CLSI | | |
| All isolates (4,400; 100) | 0.12/0.25 |  | 99.9 | 99.6 | 98.5 |  | 96.7 | 97.6 | 97.4 |
| DTR^b^ (154; 3.5) | 0.12/1 |  | 98.7 | 98.7 | 90.3 |  | 61.0 | 62.3 | 70.8 |
| BL-BLI-NS^c^ (86; 55.8) | 0.25/2 |  | 97.7 | 97.7 | 86.0 |  | 30.2 | 32.6 | 47.7 |
| CAZ-AVI-NS (60; 39.0) | 0.25/2 |  | 96.7 | 96.7 | 86.7 |  | - | 35.0 | 46.7 |
| IMI-REL-NS (58; 37.7) | 0.25/2 |  | 96.6 | 96.6 | 86.2 |  | 32.8 | - | 46.6 |
| TOL-TAZ-NS (45; 29.2) | 0.25/2 |  | 95.6 | 95.6 | 77.8 |  | 28.9 | 31.1 | - |
| CAZ-AVI/IMI-REL-NS (39; 25.3) | 0.25/2 |  | 94.9 | 94.9 | 84.6 |  | - | - | 35.9 |
| CAZ-AVI/TOL-TAZ-NS (32; 20.8) | 0.5/2 |  | 93.8 | 93.8 | 75.0 |  | - | 21.9 | - |
| IMI-REL/TOL-TAZ-NS (31; 20.1) | 0.25/2 |  | 93.5 | 93.5 | 80.6 |  | 19.4 | - | - |
| CAZ-AVI/IMI-REL/TOL-TAZ-NS (25; 16.2) | 0.5/2 |  | 92.0 | 92.0 | 76.0 |  | - | - | - |

Abbreviations: FDC, cefiderocol; CAZ-AVI, ceftazidime-avibactam; IMI-REL, imipenem-relebactam; TOL-TAZ, ceftolozane-tazobactam; DTR, difficult-to-treat; NS, nonsusceptible.

^a^ CLSI (2025), EUCAST (2025) and the FDA breakpoints for cefiderocol applied. CLSI (2025) breakpoints applied for comparator agents.

^b^ Isolates exhibiting nonsusceptible MIC results (CLSI) to piperacillin-tazobactam, ceftazidime, cefepime, aztreonam, meropenem, imipenem, ciprofloxacin, and levofloxacin.

^c^ Isolates that were nonsusceptible to ≥1 of the following BL-BLI combination agent: ceftazidime-avibactam, imipenem-relebactam, and/or ceftolozane-tazobactam.

Table S3. Activity of cefiderocol and comparator BL-BLI combinations against DTR *P. aeruginosa* collected from medical centers located in European countries, Israel and Turkey (2020-2023).

|  |  |  | % susceptible^a^ | | | | | | |
| --- | --- | --- | --- | --- | --- | --- | --- | --- | --- |
|  |  |  |  | FDC |  |  | CAZ-AVI | IMI-REL | TOL-TAZ |
| Susceptibility Profile (n; %) | MIC_50/90_ |  | CLSI | EUCAST | FDA |  | CLSI | | |
| All isolates (5,172; 100) | 0.12/0.5 |  | 99.7 | 99.4 | 98.6 |  | 95.6 | 95.5 | 94.7 |
| DTR^b^ (223; 4.3) | 0.12/2 |  | 97.8 | 95.5 | 87.9 |  | 46.2 | 51.6 | 43.0 |
| BL-BLI-NS^c^ (152; 68.2) | 0.25/2 |  | 97.4 | 94.7 | 85.5 |  | 21.1 | 28.9 | 16.4 |
| CAZ-AVI-NS (120; 53.8) | 0.25/2 |  | 97.5 | 94.2 | 82.5 |  | - | 26.7 | 14.2 |
| IMI-REL-NS (108; 48.4) | 0.25/2 |  | 97.2 | 95.4 | 87.0 |  | 18.5 | - | 10.2 |
| TOL-TAZ-NS (127; 57.0) | 0.25/2 |  | 95.9 | 94.5 | 83.5 |  | 18.9 | 23.6 | - |
| CAZ-AVI/IMI-REL-NS (88; 39.5) | 0.25/2 |  | 96.6 | 94.3 | 84.1 |  | - | - | 3.4 |
| CAZ-AVI/TOL-TAZ-NS (103; 46.2) | 0.25/2 |  | 97.1 | 94.2 | 80.6 |  | - | 17.5 | - |
| IMI-REL/TOL-TAZ-NS (97; 43.5) | 0.25/2 |  | 96.9 | 94.8 | 85.6 |  | 12.4 | - | - |
| CAZ-AVI/IMI-REL/TOL-TAZ-NS (25; 16.2) | 0.5/2 |  | 92.0 | 92.0 | 76.0 |  | - | - | - |

Abbreviations: FDC, cefiderocol; CAZ-AVI, ceftazidime-avibactam; IMI-REL, imipenem-relebactam; TOL-TAZ, ceftolozane-tazobactam; DTR, difficult-to-treat; NS, nonsusceptible.

^a^ CLSI (2025), EUCAST (2025) and the FDA breakpoints for cefiderocol applied. CLSI (2025) breakpoints applied for comparator agents.

^b^ Isolates exhibiting nonsusceptible MIC results (CLSI) to piperacillin-tazobactam, ceftazidime, cefepime, aztreonam, meropenem, imipenem, ciprofloxacin, and levofloxacin.^c^ Isolates that were nonsusceptible to ≥1 of the following BL-BLI combination agent: ceftazidime-avibactam, imipenem-relebactam, and/or ceftolozane-tazobactam.
